# Expression of an antimicrobial peptide persulcatusin fused with calmodulin in rice cultured cells

**DOI:** 10.1007/s11248-025-00449-6

**Published:** 2025-06-16

**Authors:** Gaku Fujita, So Shimoda, Minako Itagaki, Takuto Yahara, Ryuta Tobe, Hiroshi Yoneyama, Yukihiro Ito

**Affiliations:** 1 Graduate School of Agricultural Science, Tohoku University, 468-1 Aramaki Aza Aoba, Aoba-ku, Sendai, 980-8572 Japan; 2 Present Address: National Institute of Animal Health, National Agriculture and Food Research Organization, Sapporo, 062-0045 Japan

**Keywords:** Antimicrobial peptide, Persulcatusin, Molecular farming, Protein production, Rice

## Abstract

Antimicrobial proteins and peptides are potential alternatives to antibiotics. Persulcatusin is an antimicrobial peptide found in the taiga tick *Ixodes persulcatus*. We constructed fusion genes that encode, from the N-terminus to the C-terminus, a signal sequence of rice α-amylase 3D, mouse calmodulin, a target sequence of TEV protease of tobacco etch virus, and persulcatusin with or without a His tag at the N-terminus of the mature fusion protein. These fusion genes were then introduced into rice. Western blot analysis detected persulcatusin fusion proteins in transgenic calli, suspension cells, and their culture medium. Antimicrobial activity against *Staphylococcus aureus* was detected in the protein extracts prepared from the transgenic callus but not from the non-transgenic wild-type callus, and TEV protease treatment to release persulcatusin from the fusion protein enhanced antimicrobial activity. The growth of the transgenic rice plants was unaffected. Our results indicate that functional persulcatusin can be produced in rice cells. This provides a basis for the mass production of persulcatusin for therapeutic use against bacterial infectious diseases in humans and livestock.

**Supplementary Information:**

The online version contains supplementary material available at 10.1007/s11248-025-00449-6.

## Introduction

Molecular farming is a technique to produce high-quality proteins such as therapeutic proteins and vaccines (Eidenberger et al. 2023). Protein production in plants has several advantages over production in other systems, such as animal cells, yeasts, and bacteria (Obembe et al. 2011). Plants can be grown cost-effectively, and even as cultured cells where only minerals, sugars, and phytohormones, and no expensive medium such as serum or peptone generally used for animal cells or microorganisms, are necessary. The possibility of contamination with human and livestock pathogens, including unidentified viruses, is extremely low. If proteins accumulate in storage organs, such as seeds, they can be stored at room temperature. Direct intake of proteins, such as edible vaccines, is also possible if the protein of interest accumulates in the edible parts of plants. However, the amount of protein produced in plants is rather low, and plants have a glycosylation system different from that of animal cells, which may affect protein activity (Jin et al. 2025; Strasser 2023; Burnett and Burnett 2020; Schillberg et al. 2002; Chargelegue et al. 2000).

Plant protein production can be categorized into two technical groups: transient and stable. In transient expression, the gene of interest is introduced into the nucleus and expressed without the integration of the gene into chromosomes. Agroinfection is the most effective technique for transient protein production (Lindbo 2007). The gene of interest is introduced into the plant viral genome and the recombinant viral genome is placed in the T-DNA of *Rhizobium radiobacter* (*Agrobacterium tumefaciens*). Then, *Rhizobium radiobacter* is infiltrated into leaves or entire plants, and T-DNA is transferred from *Rhizobium radiobacter* to the plant nucleus, where the recombinant virus genome is transcribed and replicated, and the protein encoded by the gene of interest is translated. A large amount of protein is produced because the protein-coding recombinant viral RNA is amplified using replication. However, due to the low efficiency of infiltration, the application of agroinfection is limited to *Nicotiana benthamiana*. In addition, expensive protein purification is necessary because *N. benthamiana* produces nicotine and other alkaloids; thus, direct intake or application of crude extracts is not possible.

A foreign protein gene is integrated into the nuclear or chloroplast genome in stable expression. The chloroplast is a good target for accumulating foreign proteins because it has low proteinase activities and occurs in large numbers in the cells (Benchabane et al. 2008; Doran et al. 2006). Because chloroplast transformation is only available in a few plant species (An et al. 2022), a foreign protein gene is introduced into the nuclear genome of most species, and the foreign protein is translocated to the chloroplast by adding a chloroplast-specific transit sequence to its N-terminus (Muthamilselvan et al. 2019; Shen et al. 2017). Secretion of target proteins from suspension cells into the liquid medium is also preferred to avoid cell lysis prior to protein extraction (Arya et al. 2021). The cost of protein purification can be reduced by secretion into the culture medium. Various proteins are produced in this form in rice suspension cells (Huang et al. 2021, 2005; Liu et al. 2015; Liu 2012; Kim et al. 2008a, b; Shin et al. 2003).

A large amount of antibiotics is used for livestock production in addition to human therapeutics (Mulchandaui et al. 2023), which results in the generation and spread of antibiotic-resistant bacteria, such as methicillin-resistant *Staphylococcus aureus* (MRSA) and vancomycin-resistant *Enterococcus* (VRE) (You and Sibergeld 2014). This has become a considerable threat to public health worldwide (Cong et al. 2020; Meyer et al. 2011). The amounts of antibiotics used in livestock production and human therapeutics must be reduced to overcome this problem. Antimicrobial peptides and proteins are good candidates to replace antibiotics and reduce the use thereof (Moretta et al. 2021).

Persulcatusin (IP, Uniprot ID: B7XFT1) is an antimicrobial peptide encoded by defensin gene in the taiga tick, *Ixodes persulcatus* (Miyoshi et al. 2016; Isogai et al. 2011; Saito et al. 2009). IP showed strong antimicrobial activity against gram-positive bacteria, including MRSA and vancomycin-resistant *Staphylococcus aureus* (VRSA), and weak activity against gram-negative *Escherichia coli*. IP induces the breakage of the bacterial membrane, which is the basis of antimicrobial activity (Miyoshi et al. 2017). However, IP does not affect animal cells (Miyoshi et al. 2017). IP does not affect the growth of bovine fibroblasts or colon epithelial cells. The morphology of the IP-treated bovine colon epithelial cells was normal. IP did not damage the DNA of bovine fetal fibroblasts or bovine fetal colon epithelial cells and showed no hemolytic activity against human erythrocytes. These characteristics render IP a suitable antimicrobial agent for therapeutic use in humans and livestock. However, there have been no reports of recombinant IP production in plants.

Therefore, we examined the potential of using rice in IP production in this study.

## Results

### Generation of transgenic rice cultured cells containing persulcatusin genes

**Fig. 1:**
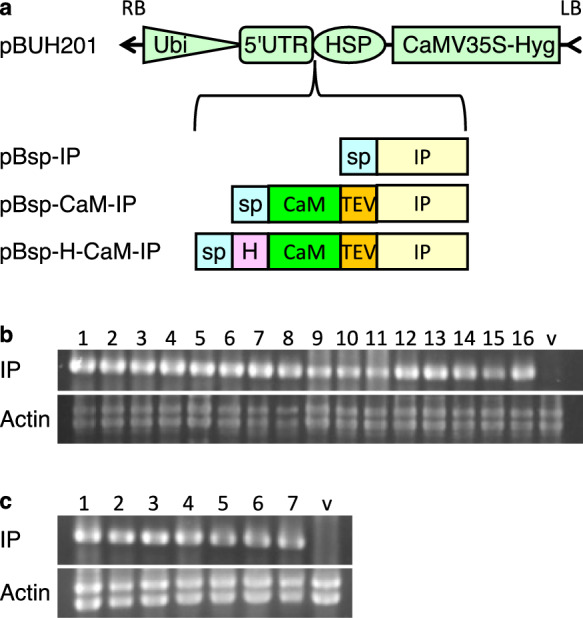
Transformation of rice. **a** Structure of vectors used for rice transformation. DNA sequences that encode signal peptide (sp)-fused persulcatusin (IP) or sp and calmodulin (CaM)-fused IP with or without 6xHis-tag (H) were inserted between 5′ untranslated region of rice alcohol dehydrogenase gene (5′UTR) and terminator of heat shock protein gene of *Arabidopsis thaliana* (HSP). These three binary vectors were used for *Rhizobium radiobacter* (*Agrobacterium*)-mediated transformation. RB and LB: right border and left border, respectively, of T-DNA, Ubi: Ubiquitin promoter, CaMV35S-Hyg: hygromycin-resistance gene driven by cauliflower mosaic virus 35S promoter, sp: signal peptide of rice α-amylase 3D, TEV: target sequence of TEV protease. **b** and **c** PCR of sp-H-CaM-IP (**b**) and sp-CaM-IP (**c**). Genomic PCR was carried out to confirm the presence of the transgenes in hygromycin-resistant calli. IP was amplified using specific primers. Actin was used as a control. Two actin bands were amplified because there are four actin genes with introns of two different lengths in the rice genome. The CaM-IP genes were detected in the transgenic calli. Lanes 1–16: 16 independent transgenic lines of sp-H-CaM-IP, lanes 1–7: 7 independent transgenic lines of sp-CaM-IP, and v: empty vector-transformed control line

We generated the binary vector pBUH201 to efficiently express persulcatusin (IP) fusion genes. pBUH201 harbors the ubiquitin promoter, 5ʹ untranslated region of rice *ADH*, *Sna*BI and *Sac*I sites for cloning, the terminator of rice *HSP* gene, and a hygromycin resistance gene in the T-DNA region (Fig. 1a). By cloning a foreign protein gene into the *Sna*BI site using In-Fusion or a similar technique, the CCATG sequence can be easily generated, where the underlined ATG is a translation start codon. It has been reported that dinucleotides immediately before the translation start codon affect translation efficiency, and CC showed high translation efficiency in rice (Sugio et al. 2010).

Binary vector plasmids were constructed for the expression of IP-fusion proteins. Because some antimicrobial peptides (AMPs) have been reported to have negative effects on plant growth (Hoelscher et al. 2022) and IP has been reported to have high antimicrobial activity (Miyoshi et al. 2017), we used the calmodulin (CaM) fusion expression system developed for AMP expression in a bacterium (Ishida et al. 2016). In this system, AMP is expressed as a fusion protein with CaM, which masks its antimicrobial activity and allows its expression and accumulation in bacterial cells. The target sequence of the TEV protease between CaM and AMP allows the release of AMP from CaM via digestion with TEV protease.

We cloned the genes encoding CaM-IP fusion proteins into the *Sna*BI site of pBUH201 (Fig. 1a). pBsp-CaM-IP encodes, from its N-terminus to C-terminus, a signal sequence of rice α-amylase 3D, mouse CaM, a target sequence of TEV protease of tobacco etch virus, and IP of taiga tick *Ixodes persulcatus* (Online Resource 1). pBsp-H-CaM-IP encodes 6xHis-tagged CaM-IP with a signal sequence at the N-terminus (Online Resource 2). The dinucleotide CC was placed immediately before the ATG translation initiation codon in these two genes.

These vectors were introduced into *Rhizobium radiobacter* and used for rice transformation. We obtained 7 and 16 hygromycin-resistant calli using pBsp-CaM-IP and pBsp-H-CaM-IP vectors, respectively. Genomic PCR analyses with transgene-specific primers confirmed the presence of transgenes in hygromycin-resistant calli (Fig. 1b and c). Each transgenic callus was separated into two pieces: one was used for expression and protein production analyses, and the other was used for the regeneration of plants to obtain self-pollinated seeds.

### Expression of persulcatusin fusion genes

**Fig. 2:**
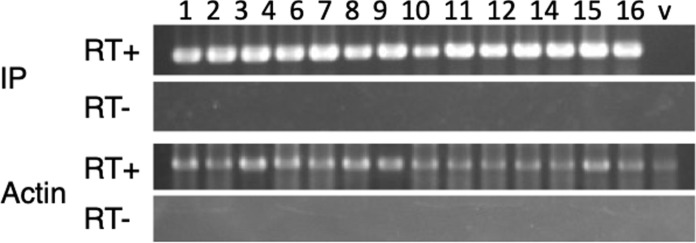
Expression of *IP* genes in transgenic rice. RT-PCR was carried out to examine the expression of sp-H-CaM-IP. Poly(A)^+^RNA was purified from total RNA isolated from transgenic calli, reverse-transcribed, and subjected to PCR using sp-H-CaM-IP-specific primers (RAmy3D-F9 and PI-R2, Table 1) or actin-specific primers (RAc-1 and RAc-2, Table 1). RT + indicates that reverse transcriptase was added to the reverse transcription reaction mixture, and RT- indicates that reverse transcriptase was omitted from the reaction mixture. Actin was used as an internal control. The transgenic calli, but not the empty vector-transformed control callus, showed the expression of CaM-IP at the RNA level. Lanes 1–16: independent transgenic calli, v: empty vector-transformed calli

Total RNA was extracted from growing calli and used for RT-PCR analysis to examine the expression of sp-H-CaM-IP and sp-CaM-IP genes. The results showed that sp-H-CaM-IP and sp-CaM-IP were expressed in all examined transgenic calli (Fig. 2; Online resource 3).

**Fig. 3:**
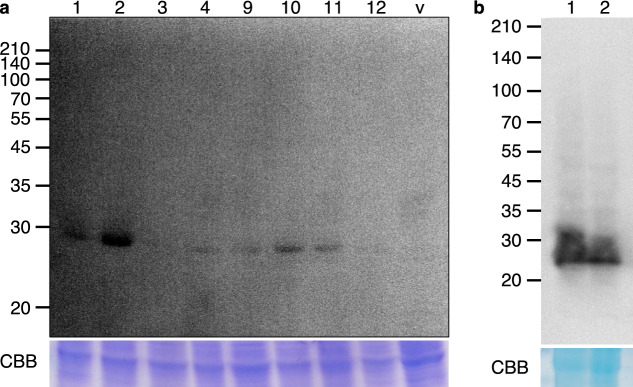
Expression of sp-H-CaM-IP and sp-CaM-IP in transgenic calli. **a** Expression of sp-H-CaM-IP. To examine the expression of sp-H-CaM-IP at a protein level western blotting was performed using protein extracts prepared from transgenic calli and an anti-His-tag antibody. The CBB-stained gel (lower panel) of the same protein extracts showing an endogenous protein was used for equal loading. Lanes 1–12: independent transgenic calli; v: empty vector-transformed calli. The numbers at the left side indicate the positions of molecular marker proteins with an indicated molecular mass. The transgenic calli, but not the empty vector-transformed control callus, showed the expression of CaM-IP at the protein level. **b** Expression of sp-CaM-IP. To examine the expression of sp-CaM-IP at a protein level western blotting was performed using protein extracts prepared from the transgenic calli and an anti-CaM antibody. The CBB-stained gel (lower panel) of the same protein extracts showing an endogenous protein was used for equal loading. Lanes 1 and 2: independent transgenic calli. The numbers at the left side indicate the positions of molecular marker proteins with an indicated molecular mass. The transgenic calli showed the expression of CaM-IP at the protein level

We examined the protein expression levels of sp-CaM-IP and sp-H-CaM-IP. Total soluble proteins were extracted from calli and subjected to western blot analysis. The results showed that bands at the expected molecular mass were detected in six out of eight sp-H-CaM-IP plants examined using an anti-His-tag antibody, and no band was detected in the empty vector-transformed control plant (Fig. 3a). Bands were also detected in the protein extracts prepared from sp-CaM-IP plants using an anti-CaM antibody (Fig. 3b). These results indicated that sp-H-CaM-IP and sp-CaM-IP were produced in the transgenic rice calli.

**Fig. 4:**
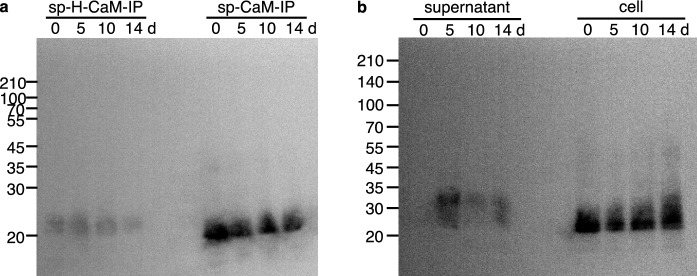
Expression of sp-H-CaM-IP and sp-CaM-IP in transgenic suspension cells. **a** Expression of sp-H-CaM-IP and sp-CaM-IP in suspended cells. To examine the expression of CaM-IP fusion proteins in suspension cells western blotting was performed using protein extracts prepared from transgenic suspension cells of pBsp-H-CaM-IP and pBsp-CaM-IP at 0, 5, 10, and 14 days after transfer to fresh culture medium. An anti-CaM antibody was used to detect the proteins. The numbers at the left side indicate the positions of molecular marker proteins with an indicated molecular mass. The transgenic suspension cells showed the expression of CaM-IP at the protein level. **b** Accumulation of sp-CaM-IP in the culture medium. To examine the secretion and accumulation of CaM-IP in the culture medium western blotting was performed using liquid culture medium (supernatant) and protein extracts from the suspension cells (cell) of pBsp-CaM-IP at 0, 5, 10, and 14 days after transfer to fresh culture medium. An anti-CaM antibody was used to detect the proteins. The numbers at the left side indicate the positions of molecular marker proteins with an indicated molecular mass. CaM-IP was detected both in the suspension cells and their culture medium (supernatant)

We also examined the expression of IP fusion proteins in suspension cells. Protein extracts were prepared from pBsp-H-CaM-IP #2 and pBsp-CaM-IP #1 at 0, 5, 10, and 14 days after transfer (DAT) to fresh medium. Bands with similar intensities from both sp-H-CaM-IP and sp-CaM-IP on all examined days were detected using western blot analysis with an anti-CaM antibody (Fig. 4a).

### Secretion of sp-CaM-IP into the culture medium

Since sp-CaM-IP has a signal sequence at its N-terminus and is expected to be secreted into the culture medium, we examined the accumulation of sp-CaM-IP in the culture medium. Culture media of pBsp-CaM-IP #1 at 0, 5, 10, and 14 DAT were used for western blot analysis. A strong band was detected at 5 DAT, whereas only weak bands were detected at 10 and 14 DATs. No bands were detected in the medium immediately after transfer (0 DAT) (Fig. 4b). The suspension cells maintained a similar expression level throughout culturing (Fig. 4b). These results indicated that sp-CaM-IP was expressed in suspension cells and secreted into the culture medium, where sp-CaM-IP was degraded. In addition, the position of the bands detected in the culture medium shifted to an upper position compared to that of the suspension cells (Fig. 4b). This suggests protein modification including glycosylation of sp-CaM-IP.

### Expression level of the sp-CaM-IP and sp-H-CaM-IP in the next generation

**Fig. 5:**
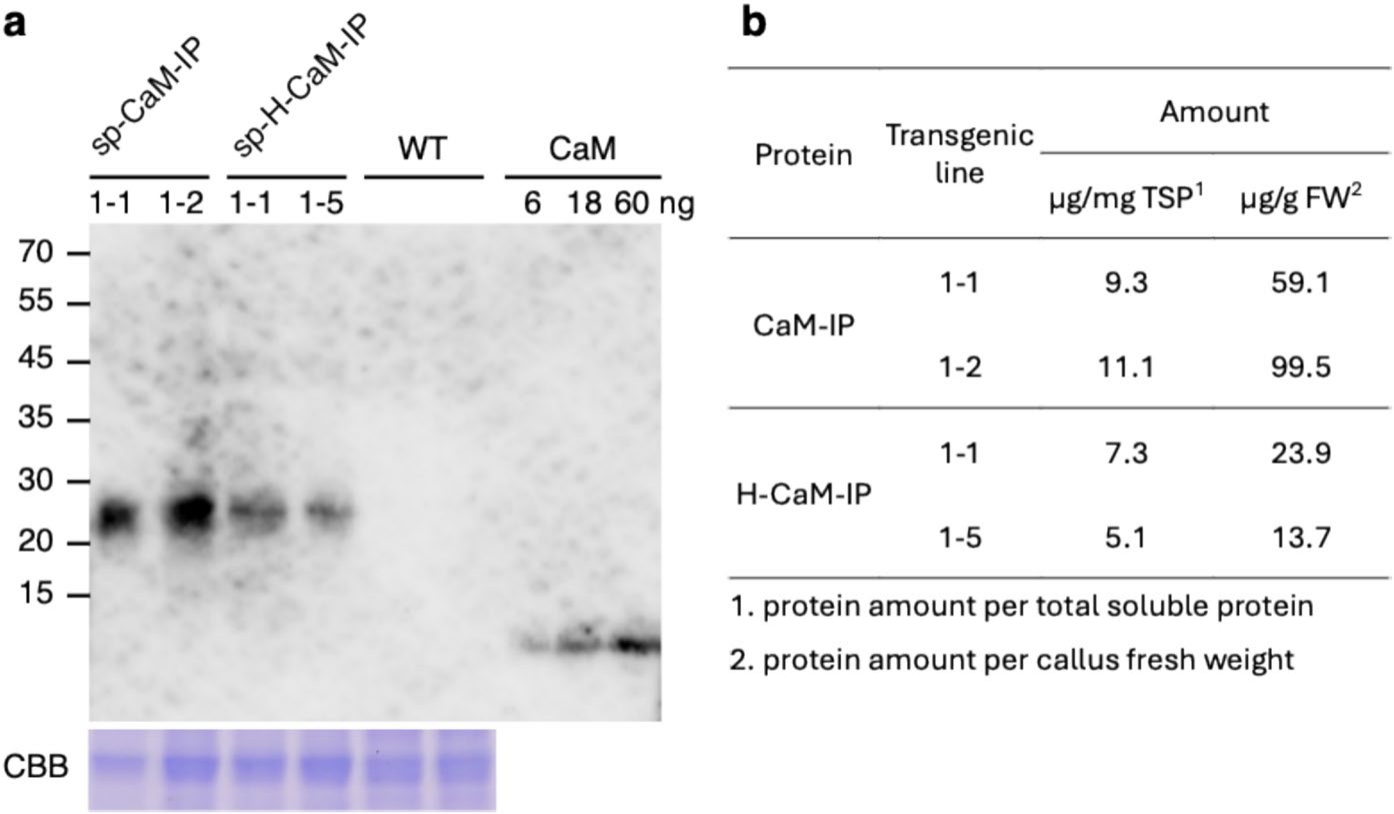
Expression level of sp-CaM-IP and sp-H-CaM-IP in calli induced from a self-progeny seed. **a** Expression of sp-CaM-IP and sp-H-CaM-IP. To examine the expression level of sp-CaM-IP and sp-H-CaM-IP western blotting was performed using protein extracts (12 μg) prepared from the calli induced from self-progeny seeds or wild type seeds. Calmodulin (6 ng, 18 ng and 60 ng in each lane) was also loaded as a standard. An anti-CaM antibody was used to detect CaM-IP fusion proteins. The CBB-stained gel (lower panel) of the same protein extracts showing an endogenous protein was used for equal loading. The numbers at the left side indicate the positions of molecular marker proteins with an indicated molecular mass. The transgenic calli showed the expression of CaM-IP at the protein level. **b** The amount of sp-CaM-IP and sp-H-CaM-IP. The intensity of the bands of **a** was measured with Image J, and the amount of the IP fusion proteins were calculated using calmodulin (60 ng) as a standard

Self-pollinated progeny seeds of sp-CaM-IP plants and sp-H-CaM-IP were obtained from regenerated plants, and calli were induced from the seeds to examine the expression level of IP fusion proteins by western blot probed with an anti-calmodulin antibody. Calmodulin was used as a standard to calculate the amount. Measurement of band intensity and comparison with that of calmodulin showed that sp-CaM-IP calli produced 9.3 μg and 11.1 μg per mg of the total soluble protein, and sp-H-CaM-IP callus produced 7.3 μg and 5.1 μg (Fig. 5). Protein production per biomass (callus fresh weight) was 59.1 μg and 99.5 μg for sp-CaM-IP, and 23.9 μg and 13.7 μg for H-CaM-IP (Fig. 5). We also estimated the amount of sp-CaM-IP in calli using additional pBsp-CaM-IP lines. The maximum amount of spCaM-IP was 34.9 μg per mg of the total soluble protein (Online Resource 4).

### Antimicrobial activities of persulcatusin produced in rice

**Fig. 6:**
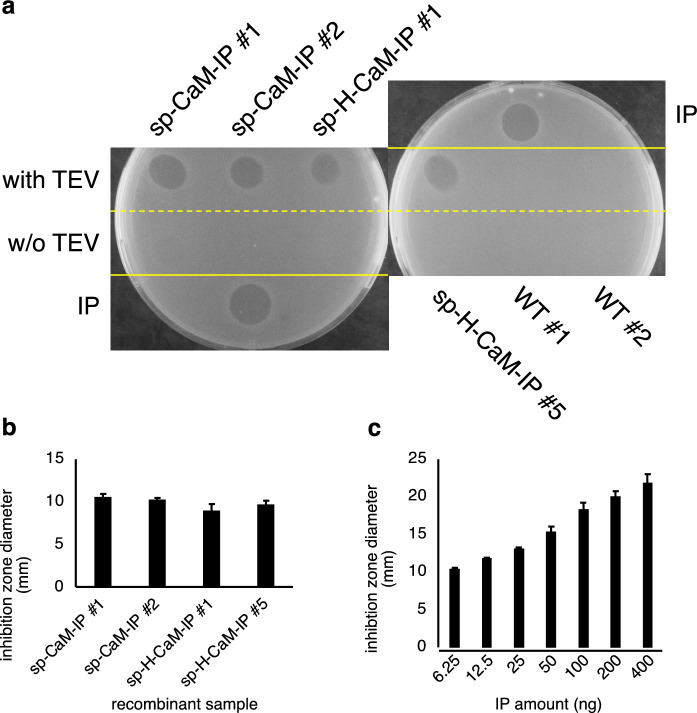
An antimicrobial activity of sp-CaM-IP and sp-H-CaM-IP against *Staphylococcus aureus*. **a** Growth inhibition assay. To examine an antimicrobial activity of CaM-IP fusions and IP protein extracts prepared from calli expressing sp-CaM-IP, sp-H-CaM-IP, or the control wild-type callus were spotted on the indicator *S. aureus* cell-impregnated agar medium. TEV-treated protein extracts (with TEV) were also spotted in addition to untreated protein extracts (w/o TEV). After incubation for 3 h at room temperature, the plates were covered with an overlay medium. Next, the growth levels of the indicator strain are observed after incubation at 30 °C for 20 h. An antimicrobial activity was observed in the TEV-treated protein extracts from the transgenic calli, but no activity was observed in the untreated extracts or the WT extracts. IP indicates a spot of chemically synthesized IP. **b** and **c** Measurement of an antimicrobial activity. The diameter of the inhibition zone of IP fusion proteins (**b**) and chemically synthesized IP (**c**) was measured. The average and standard error of the inhibition zones were calculated using the data of three replicates. No significant difference was observed among the samples (Mann–Whitney *U* test, *P* < 0.05, Online Resource 8)

We performed a radial diffusion assay against the gram-positive bacterium *Staphylococcus aureus* to examine the antimicrobial activity of IP produced in rice. Protein extracts were prepared from calli of sp-CaM-IP and sp-H-CaM-IP and incubated with TEV protease, which specifically digests the target sequence between CaM and IP. These protein extracts were spotted on the medium, and bacterial growth was examined. We observed an inhibition zone around the position where the TEV-treated protein extracts from sp-CaM-IP and sp-H-CaM-IP calli were spotted, whereas inhibition zone was barely observed around the position where the non-treated protein extracts or the protein extracts from non-transgenic calli were spotted (Fig. 6).

We repeated a radial diffusion assay, and protein extracts prepared from suspension cells and their culture medium were also examined. We reproducibly observed an inhibition zone around the position where the TEV-treated protein extracts from sp-CaM-IP and sp-H-CaM-IP calli were spotted, and, interestingly, the TEV-untreated callus extracts showed weaker anti-*S. aureus* activities compared to the TEV-treated samples (Online Resource 5). In addition, the culture medium of sp-CaM-IP suspension cells showed weak activities, indicating that CaM-IP was secreted from the cells as expected (Online Resource 5). Inhibition region was barely observed around the position where the protein extracts from non-transgenic wild type calli were spotted.

These results indicated that IP produced in rice has an antimicrobial activity against *S. aureus* and that CaM fusion mostly masks the antimicrobial activity of IP.

### Effects of IP expression on rice growth

Because some antimicrobial peptides have been reported to affect plant growth negatively (Hoelscher et al. 2022) and IP has been reported to have high antimicrobial activity (Miyoshi et al. 2017), we examined the effects of IP expression on plant growth. We generated another transgenic rice plant that expressed IP alone, without the CaM fusion, but with a signal sequence at its N-terminus (sp-IP) (Fig. 1a). RT-PCR confirmed the expression of sp-IP (Online Resource 6). We compared the growth, morphology, and seed setting of plants expressing sp-H-CaM-IP and sp-IP alone with those of control plants. We found no differences between the three plants under normal growth conditions (Online Resource 7). This suggested that the IP does not affect rice growth, morphology, or seed setting.

## Discussion

In this study, we generated transgenic rice cells that produced IP fusion proteins. sp-CaM-IP and sp-H-CaM-IP produced in rice showed antimicrobial activity against *S. aureus*. The expression of sp-H-CaM-IP and sp-IP did not affect the plant growth. These results indicated that rice can be used as a platform for IP production.

The signal sequence of rice amylase was fused to the N-terminus of the IP fusions to be secreted into the culture medium when expressed in suspension cells; as expected, sp-CaM-IP was detected in the medium (Fig. 4b). However, the amount of sp-CaM-IP accumulated in the medium peaked at 5 DAT and decreased after 10 DAT, even when sp-CaM-IP was continuously expressed in the suspension cells. This suggested that CaM-IP was degraded in the medium by secreted proteases. The secretion of proteases from rice suspension cells into the culture medium has been suggested (Kim et al. 2007, 2008b). Because proteins secreted into the medium from rice suspension cells are very limited, the secretion of a useful protein is an ideal method to reduce the labor and cost of protein purification. Using a knockout line of the proteinase gene or the expression of a proteinase inhibitor gene is a possible approach to overcome the degradation of the target proteins. It is reported that the expression of NbPR4, NbPot1, and HsTIMP, which inhibit cysteine, serine, and metalloproteases, respectively, enhanced the accumulation of α-galactosidase, erythropoietin, and IgG antibody in the transient expression system of *Nicotiana benthamiana* (Grosse-Holz et al. 2018). In rice suspension cells, the expression of synthetic genes encoding the chymotrypsin and trypsin inhibitor domains of *Nicotiana alata* serine proteinase inhibitor II enhanced the accumulation of human granulocyte–macrophage colony-stimulating factor (Kim et al. 2008b). Thus, the expression of a protease inhibitor gene or knockout of the protease gene is required for high accumulation of IP fusion proteins in the culture medium of rice suspension cells.

By comparing the sp-CaM-IP extracted from the cell suspension and that in the culture medium, it appeared that a large portion of sp-CaM-IP remained in the cells without being secreted. We used a signal sequence of rice α-amylase 3D because it is highly expressed in the cultured cells in this study (Huang et al. 1993). Its signal sequence successfully secreted several proteins such as human serum albumin, human octamer-binding transcription factor 4 (Oct4), human granulocyte–macrophage colony-stimulating factor (hGM-CSF) from rice suspension cells (Huang et al. 2021, 2005; Shin et al. 2003). It has been reported that the efficiency of protein secretion into the medium depends on the combination of the signal sequence and the protein to be secreted (Huang et al. 2015). It is necessary to search for a signal sequence that efficiently secretes CaM-IP or IP alone into the liquid culture medium for accumulation in the medium.

In this study, IP was expressed as a fusion with CaM. The fusion of CaM to an antimicrobial peptide (AMP) has been reported to mask antimicrobial activity, enabling the production of AMP in bacterial cells such as *E. coli* (Ishida et al. 2016). In this system, the recognition sequence of the TEV protease was inserted between CaM and AMP so that after production of the CaM-AMP fusion, AMP can be released from CaM by proteolytic digestion by TEV protease, and AMP can recover its full antimicrobial activity. Consistent with this, sp-CaM-IP produced in rice showed higher antimicrobial activity after treatment with TEV protease than non-treated sp-CaM-IP. This indicated that the system can function in rice plants. This further suggested that an AMP with a negative effect on rice cells can be produced in rice cells by fusion with CaM, and the CaM-AMP fusion can be activated by digestion with TEV protease.

The expression of sp-IP alone without the CaM fusion showed no negative effect on rice growth. Thus, the simple and efficient method to produce IP in rice is to express sp-IP alone, which is then secreted to the medium. However, although the expression of sp-IP was detected at an RNA level by RT-PCR, the expression at a protein level was not examined due to a lack of IP-specific antibody. In addition, it is not known whether IP with other subcellular localization such as chloroplasts or endoplasmic reticulum, which are often targeted for recombinant protein production in plants, gives no negative effect on rice growth. Further studies are necessary to develop this simple and efficient method.

The H-CaM-IP expression system in rice suspension cells has an advantage in its purification. A culture medium of rice suspension cells contains only a small amount of proteins including amylase (Huang et al. 2015). Thus, if CaM-IP is secreted to the medium, its purification cost may be reduced. Addition of His-tag to a recombinant protein simplifies the purification, and His-tagged CaM-IP (H-CaM-IP) can be easily purified using Ni-resin, Then, H-CaM-IP bound to Ni-resin is digested by TEV protease to release IP. TEV protease can be removed by capturing to Ni-resin, if His-tagged TEV protease is used.

In the inhibition assay to detect an antimicrobial activity of recombinant IP produced by the transgenic rice cells, we used crude extracts treated with TEV protease. Although our results showed that rice-produced recombinant IP has an antimicrobial activity against *S. aureus*, the amount of released IP from the CaM fusions is not known. In addition, it is also not known whether endogenous rice proteins in the extracts give positive or negative effects or no effect on the activity. Protein purification and detection of released IP by its specific antibody will be necessary for more precise estimation of its antimicrobial activity in further studies.

In conclusion, we generated transgenic rice calli and suspension cells that express persulcatusin as a fusion protein. Rice-produced CaM-IP treated with TEV protease to release IP from CaM showed antimicrobial activity against *Staphylococcus aureus*. IP can be used as a novel therapeutic agent against infection of a gram-positive bacterial pathogen. Our results provide a fusion technology to produce an AMP that is toxic to the host plant.

## Materials and methods

### Vector construction

The binary vector pBUH201 was generated by modifying the pRI201-ON vector (Takara Bio, Kusatsu, Japan). Cauliflower mosaic virus 35S promoter and a coding sequence of hygromycin resistance gene were amplified using PCR using pBUH3 (Nigorikawa et al. 2012) as a template, and the PCR product was digested with *Kpn*I and *Apa*I, whose restriction sites were added to the 5ʹ end of the forward and reverse primers. This PCR fragment was cloned into pRI201-ON that was digested with the same restriction enzymes to replace the kanamycin-resistance gene with a hygromycin-resistance gene. The resulting plasmid was named pBCH201. The maize ubiquitin promoter was amplified using PCR from pBUH3, and the 5ʹ untranslated region (5ʹ UTR) of the rice *ADH* gene was amplified from pRI201-ON, and these two PCR fragments were ligated using a second round of PCR. The resultant PCR fragment contained a *Hind*III site, the ubiquitin promoter, the *ADH* 5ʹ UTR, and the *Sna*BI and *Sac*I sites in this order. The *Hind*III site was added to the 5ʹ end of the forward primer, and the *Sac*I and *Sna*BI sites were added to the 5ʹ end of the reverse primer. This PCR product was digested with *Hind*III and *Sac*I and cloned into pBCH201 digested with the same restriction enzymes. The resultant binary vector, named pBUH201, had a ubiquitin promoter for the expression of the gene of interest, *Sna*BI and *Sac*I sites for cloning, and a hygromycin resistance gene as a selection marker gene in the T-DNA region. Nucleotide sequences of PCR-amplified regions were verified using sequencing (the ubiquitin promoter and ADH 5ʹ UTR) or functionality (the hygromycin resistance gene) in *Rhizobium radiobacter*.

Artificial gene synthesis of sp-CaM-IP and sp-H-CaM-IP, with codons adjusted for expression in rice, was performed by Eurofins Genomics K. K. (Tokyo, Japan). sp-CaM-IP encodes a protein with a signal sequence of rice α-amylase 3D, mouse calmodulin (CaM), a target sequence of TEV proteinase of tobacco etch virus, and persulcatusin of *I. persulcatus* from its N-terminus- to C-terminus. sp-H-CaM-IP encodes a protein with a His-tag at the N-terminus of the mature sp-CaM-IP. These two genes were amplified using PCR and cloned into the *Sna*BI site of pBUH201 using In-Fusion (Takara Bio). The signal sequence and IP were amplified from sp-CaM-IP using PCR and cloned into pBUH201 using In-Fusion (Takara Bio) to generate a binary vector plasmid for expression of IP alone with the signal sequence (sp-IP). The nucleotide sequences amplified using PCR were verified using sequencing.

### Rice transformation and tissue culture

The binary vector plasmid was introduced into *Rhizobium radiobacter* EHA101 via electroporation. *Rhizobium radiobacter* -mediated transformation of rice (*Oryza sativa* Nipponbare) was performed as described by Hiei et al. (1994). Surface-sterilized seeds were put onto N6CI medium (N6 salts and vitamins, 300 mg/L casamino acid, 2827 mg/L proline, 2 mg/L 2,4-dichlorophenoxyacetic acid (2,4-D), 30 g/L sucrose, pH5.8) and incubated at 28 °C for 3 weeks. After infection of *Rhizobium radiobacter* on N6CO medium (N6 salts and vitamins, 2 mg/L 2,4-D, 30 g/L sucrose, 10 g/L glucose, 40 mg/L acetosyringone, pH5.2) at 28 °C for 3 days, transformed calli were transfer onto N6SE medium (N6 salts and vitamins, 2 mg/L 2,4-D, 30 g/L sucrose, 50 mg/L hygromycin, 40 mg/L meropen (Sumitomo Pharma, Osaka, Japan), pH5.8) and incubated for 3 weeks followed by transfer to fresh N6SE medium and additional 3-week incubation. Each hygromycin-resistant callus was separated into two pieces, one of which was used for shoot regeneration on MSRE medium (MS salts and vitamins, 1 mg/L 1-naphthylacetic acid (NAA), 2 mg/L 6-benzyladenine (BA), 2 g/L casamino acid, 30 g/L sucrose, 30 g/L sorbitol, pH5.8) with transfer onto fresh MS medium every 3 weeks for several times followed by transfer onto MSHF medium (MS salts and vitamins, 30 g/L sucrose, 50 mg/L hygromycin, 25 mg/L meropen, pH5.8) for rooting in order to harvest self-pollinated seeds, and the other was maintained as a callus for further analyses. The callus was maintained on N6CI medium at 28 °C by transferring it to fresh N6CI medium every three weeks. Calli were incubated in 30 mL of liquid N6CI medium at 28 °C with shaking at 100 rpm to generate suspension cells. Subsequently, 3 mL of the medium containing suspension cells were transferred to 27 mL of fresh medium every two weeks.

### Genomic PCR analysis

Genomic DNA was isolated from calli using a CTAB method. PCR analysis was conducted using specific primers (Table 1) and genomic DNA as the template. The following cycling parameters were used: 30 cycles of denaturation at 94 °C for 30 s, annealing at 60 °C for 30 s, and extension at 72 °C for 30 s, followed by incubation at 72 °C for 5 min. The PCR products were subjected to electrophoresis.

**Table 1:** Primers used in this study

Primer	Sequence	Target	Purpose
HPT-ZF	GAGAGCCTGACCTATTGCAT	Hygromycin phosphotransferase	Genomic PCR
HPT-ZR	TCGGCGAGTACTTCTACACA	Hygromycin phosphotransferase	Genomic PCR
PI-F1	GGGTTTGGCTGCCCTTTCAA	Persulcatusin	RT-PCR
PI-R1	TCATCTGGAATAGCACGTGC	Persulcatusin	Vector construction
PI-R2	ACCTCCACGTCTGCCAATGC	Persulcatusin	Genomic PCR, RT-PCR
R-PI-pBUH	TCATAAGAGCTCTACTCATCTGGAATAGCACGTG	Persulcatusin	RT-PCR
RAc-1	AACTGGGATGATATGGAGAA	Actin	Genomic PCR, RT-PCR
RAc-2	CCTCCAATCCAGACACTGTA	Actin	Genomic PCR, RT-PCR
RAmy3D-F9	CATGAAGAACACCTCGTCAC	Amylase 3D	Vector construction Genomic PCR, RT-PCR

### RT-PCR analysis

Total RNA was isolated from the calli using the guanidine-thiocyanate method. Poly(A)^+^RNA was purified from total RNA using Dynabeads (Thermo Fisher Scientific, Waltham, MA, USA). Half of the poly(A)^+^RNA was reverse transcribed using Superscript III Reverse Transcriptase (Thermo Fisher Scientific) or PrimeScript II Reverse Transcriptase (Takara Bio), and the remaining half was treated similarly without reverse transcriptase. PCR using specific primers (Table 1) was conducted using 30 cycles of denaturation at 94 °C for 30 s, annealing at 60 °C for 30 s, and extension at 72 °C for 30 s, followed by 5 min incubation at 72 °C. The PCR products were subjected to electrophoresis.

### Western blot analysis

Total soluble proteins were extracted from calli and cell suspensions in TBS buffer. The protein concentration was determined using the Bradford method (Bio-Rad, Hercules, CA, USA), and BSA was used as a standard. Protein extracts were stored at − 20 °C until use. Briefly, 10 μg protein or 10 μL culture medium was subjected to SDS-PAGE, blotted onto PVDF membrane, and reacted with an antibody in TBS containing 1% BSA and 0.5% Tween-20. An anti-His-tag antibody (3000-fold dilution) (Cell Signaling Technology, Danvers, MA, USA) or an anti-CaM antibody (15,000-fold dilution) (Abcam, Cambridge, UK) was used as the primary antibody, and an alkaline phosphatase-conjugated anti-rabbit IgG antibody (3000-fold dilution) (Promega, Madison, WI, USA) was used as a secondary antibody. Disodium 3-(4-methoxyspiro {1,2-dioxetane-3,2′-(5′-chloro)tricyclo [3.3.1.13,7]decan}-4-yl)phenyl phosphate (CSPD) (Roche, Basel, Switzerland) was used as a substrate of AP, and signals were detected with Image Quant LAS 4000 (GE Healthcare, Chicago, IL, USA) or ChemiDoc Imaging System (Bio-Rad).

### Estimation of the amount of IP fusion proteins

Western blot was carried out as described above using 12 μg total soluble protein and an anti-CaM antibody (Abcam). Bovine calmodulin (Fuji Film Wako Pure Chemical, Osaka Japan) was used as a standard. Band intensity was measured with Image J. Signal intensity just above or below each band was subtracted for compensation of background. The protein amount was calculated based on the band intensity and compensated with their molecular mass.

### Detection of antimicrobial activity

For digestion of CaM-IP with TEV protease, 5 μL or 10 μL of TEV protease (Nippon Gene, Tokyo, Japan) was added to 100 μL or 200 μL of the protein extract, respectively, and the reaction mixture was incubated at 4 °C overnight.

The radial diffusion method reported by Lehrer et al. (1991) was used in this study to detect antimicrobial activity. Briefly, *S. aureus* 209p (https://bacdive.dsmz.de/strain/14445) was grown in Trypticase soy broth (Nissui, Tokyo, Japan) overnight at 30 °C. The bacterial cells were centrifuged at 5000 × *g* for 5 min at room temperature (around 25 °C), washed with 10 mM sodium phosphate buffer (pH 7.4), and resuspended in the same buffer at an optical density of 0.1 at 660 nm (approximately 5.0 × 10^7^ cfu/mL). Subsequently, 1 mL of the bacterial suspension was mixed with 10 mL of pre-warmed (50 °C) underlay-medium (0.3% (w/v) TSB, 10 mM sodium phosphate buffer (pH 7.4), 1% (w/v) of low-electroendosmosis type agarose (Bio-rad), and a final concentration of 0.02% (v/v) Tween 20 (Nacalai Tesque, Kyoto, Japan) and 10 mL of the mixture was quickly poured into a culture dish. Protein extract or enzymatic reaction mixtures (5 μL) were repeatedly spotted on the surface of the hardened medium (total sample volume, 20 μL). Chemically synthesized IP (Scrum, Tokyo, Japan) with the indicated amount was also spotted. The plates were incubated for 3 h at room temperature, and then the overlaid medium (6% (w/v) TSB, 10 mM sodium phosphate buffer (pH 7.4), and 1% (w/v) agarose) was poured onto the plates. After incubation at 30 °C for 20 h, the inhibition zones were measured. The average and standard error of the inhibition zones were calculated using the data of three replicates.

### Statistic analysis

Sharpiro-Wilk test was carried out to examine normal distribution (*P* < 0.05), and since the prerequisite was not met, Mann–Whitney *U* test was carried out as a significance test at *P* < 0.05. The number of the samples of synthesized IP was 3, and the inhibition assay of the rice extracts was repeated three times.

## Supplementary Information

Below is the link to the electronic supplementary material.

Supplementary file1 (PPTX 52 kb)

Supplementary file2 (PPTX 53 kb)

Supplementary file3 (PPTX 143 kb)

Supplementary file4 (PPTX 1109 kb)

Supplementary file5 (PPTX 226 kb)

Supplementary file6 (PPTX 1247 kb)

Supplementary file7 (PPTX 9550 kb)

Supplementary file8 (XLSX 10 kb)

## Data Availability

Data sharing is not applicable to this article, as no datasets were generated or analyzed in the current study.
