# Supplementary material for: Expression of an antimicrobial peptide persulcatusin fused with calmodulin in rice cultured cells
Source: Transgenic Res. 2025 Jun 16;34(1):30. doi: 10.1007/s11248-025-00449-6 (PMC12170776; doi:10.1007/s11248-025-00449-6)
Supplement: Supplementary file 1 — Supplementary file1 (PPTX 52 kb) [file 11248_2025_449_MOESM1_ESM.pptx]

## Slide 1
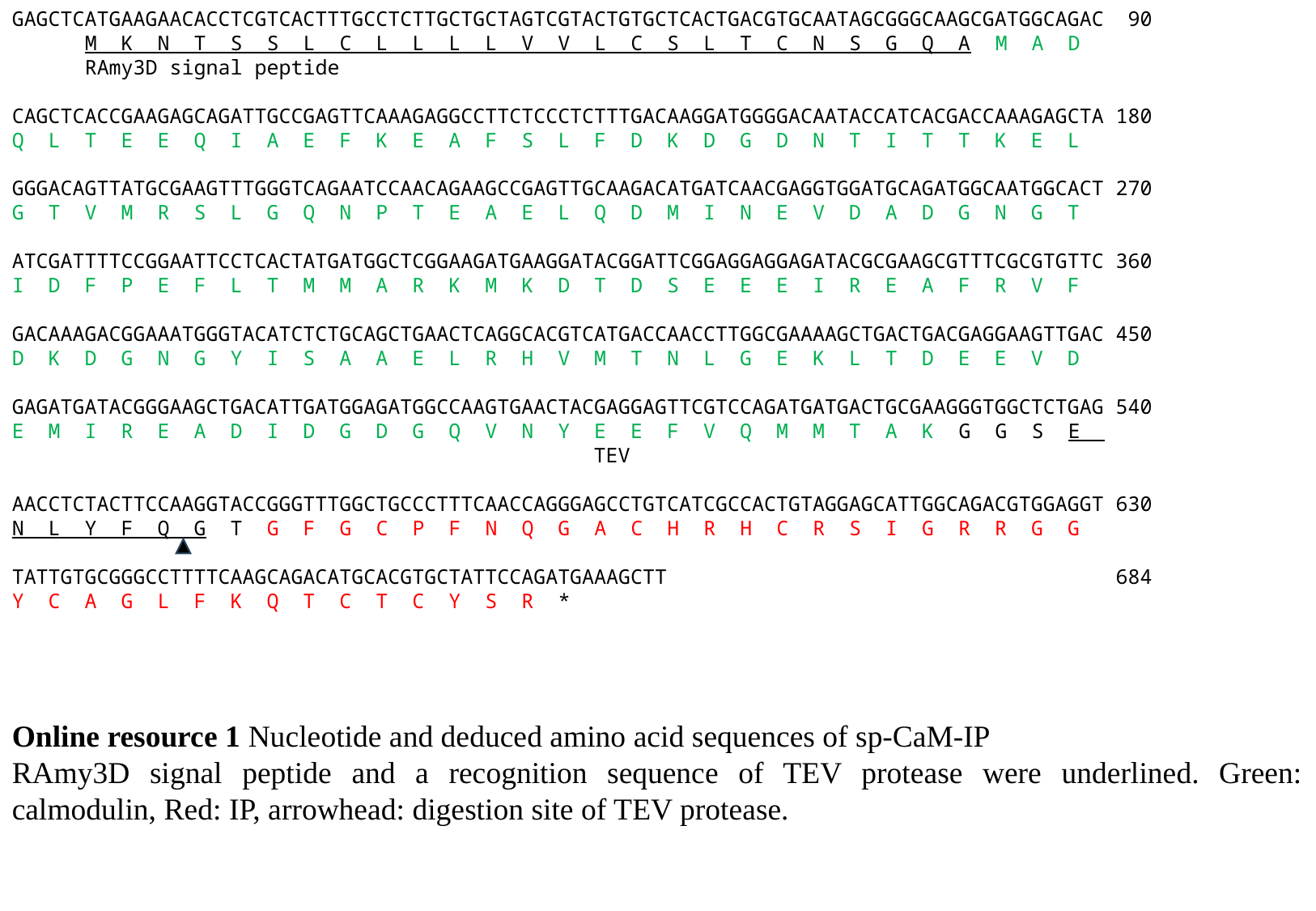

GAGCTCATGAAGAACACCTCGTCACTTTGCCTCTTGCTGCTAGTCGTACTGTGCTCACTGACGTGCAATAGCGGGCAAGCGATGGCAGAC 90
 M K N T S S L C L L L L V V L C S L T C N S G Q A M A D
 RAmy3D signal peptide
CAGCTCACCGAAGAGCAGATTGCCGAGTTCAAAGAGGCCTTCTCCCTCTTTGACAAGGATGGGGACAATACCATCACGACCAAAGAGCTA 180
Q L T E E Q I A E F K E A F S L F D K D G D N T I T T K E L
GGGACAGTTATGCGAAGTTTGGGTCAGAATCCAACAGAAGCCGAGTTGCAAGACATGATCAACGAGGTGGATGCAGATGGCAATGGCACT 270
G T V M R S L G Q N P T E A E L Q D M I N E V D A D G N G T
ATCGATTTTCCGGAATTCCTCACTATGATGGCTCGGAAGATGAAGGATACGGATTCGGAGGAGGAGATACGCGAAGCGTTTCGCGTGTTC 360
I D F P E F L T M M A R K M K D T D S E E E I R E A F R V F
GACAAAGACGGAAATGGGTACATCTCTGCAGCTGAACTCAGGCACGTCATGACCAACCTTGGCGAAAAGCTGACTGACGAGGAAGTTGAC 450
D K D G N G Y I S A A E L R H V M T N L G E K L T D E E V D
GAGATGATACGGGAAGCTGACATTGATGGAGATGGCCAAGTGAACTACGAGGAGTTCGTCCAGATGATGACTGCGAAGGGTGGCTCTGAG 540
E M I R E A D I D G D G Q V N Y E E F V Q M M T A K G G S E x
 　　　　　　　　TEV
AACCTCTACTTCCAAGGTACCGGGTTTGGCTGCCCTTTCAACCAGGGAGCCTGTCATCGCCACTGTAGGAGCATTGGCAGACGTGGAGGT 630
N L Y F Q G T G F G C P F N Q G A C H R H C R S I G R R G G
TATTGTGCGGGCCTTTTCAAGCAGACATGCACGTGCTATTCCAGATGAAAGCTT 684
Y C A G L F K Q T C T C Y S R *
Online resource 1 Nucleotide and deduced amino acid sequences of sp-CaM-IP
RAmy3D signal peptide and a recognition sequence of TEV protease were underlined. Green: calmodulin, Red: IP, arrowhead: digestion site of TEV protease.
