# Supplementary material for: Expression of an antimicrobial peptide persulcatusin fused with calmodulin in rice cultured cells
Source: Transgenic Res. 2025 Jun 16;34(1):30. doi: 10.1007/s11248-025-00449-6 (PMC12170776; doi:10.1007/s11248-025-00449-6)
Supplement: Supplementary file 2 — Supplementary file2 (PPTX 53 kb) [file 11248_2025_449_MOESM2_ESM.pptx]

## Slide 1
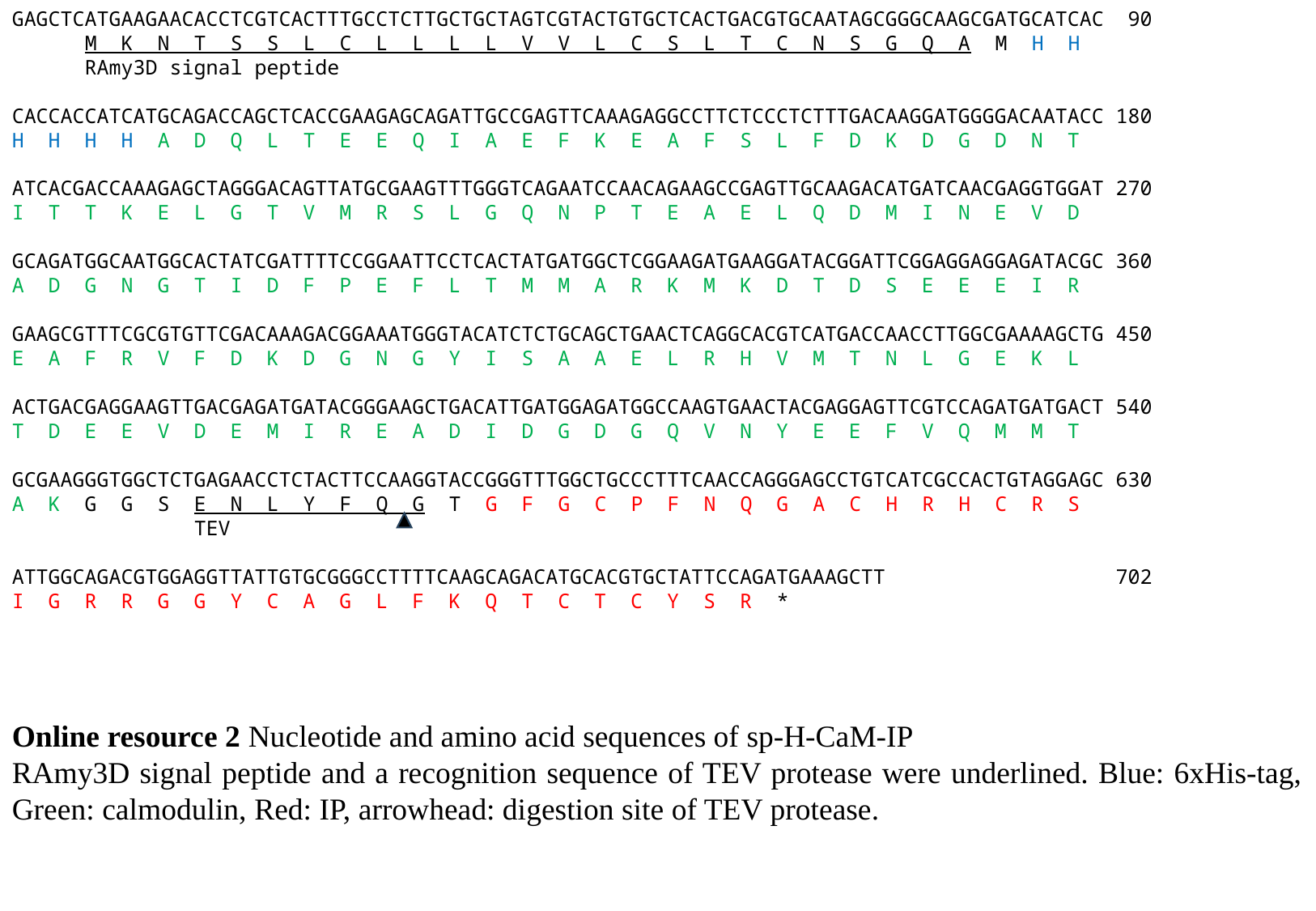

GAGCTCATGAAGAACACCTCGTCACTTTGCCTCTTGCTGCTAGTCGTACTGTGCTCACTGACGTGCAATAGCGGGCAAGCGATGCATCAC 90
 M K N T S S L C L L L L V V L C S L T C N S G Q A M H H
 RAmy3D signal peptide
CACCACCATCATGCAGACCAGCTCACCGAAGAGCAGATTGCCGAGTTCAAAGAGGCCTTCTCCCTCTTTGACAAGGATGGGGACAATACC 180
H H H H A D Q L T E E Q I A E F K E A F S L F D K D G D N T
ATCACGACCAAAGAGCTAGGGACAGTTATGCGAAGTTTGGGTCAGAATCCAACAGAAGCCGAGTTGCAAGACATGATCAACGAGGTGGAT 270
I T T K E L G T V M R S L G Q N P T E A E L Q D M I N E V D
GCAGATGGCAATGGCACTATCGATTTTCCGGAATTCCTCACTATGATGGCTCGGAAGATGAAGGATACGGATTCGGAGGAGGAGATACGC 360
A D G N G T I D F P E F L T M M A R K M K D T D S E E E I R
GAAGCGTTTCGCGTGTTCGACAAAGACGGAAATGGGTACATCTCTGCAGCTGAACTCAGGCACGTCATGACCAACCTTGGCGAAAAGCTG 450
E A F R V F D K D G N G Y I S A A E L R H V M T N L G E K L
ACTGACGAGGAAGTTGACGAGATGATACGGGAAGCTGACATTGATGGAGATGGCCAAGTGAACTACGAGGAGTTCGTCCAGATGATGACT 540
T D E E V D E M I R E A D I D G D G Q V N Y E E F V Q M M T
GCGAAGGGTGGCTCTGAGAACCTCTACTTCCAAGGTACCGGGTTTGGCTGCCCTTTCAACCAGGGAGCCTGTCATCGCCACTGTAGGAGC 630
A K G G S E N L Y F Q G T G F G C P F N Q G A C H R H C R S
 TEV
ATTGGCAGACGTGGAGGTTATTGTGCGGGCCTTTTCAAGCAGACATGCACGTGCTATTCCAGATGAAAGCTT 702
I G R R G G Y C A G L F K Q T C T C Y S R *
Online resource 2 Nucleotide and amino acid sequences of sp-H-CaM-IP
RAmy3D signal peptide and a recognition sequence of TEV protease were underlined. Blue: 6xHis-tag, Green: calmodulin, Red: IP, arrowhead: digestion site of TEV protease.
