# Supplementary material for: Expression of an antimicrobial peptide persulcatusin fused with calmodulin in rice cultured cells
Source: Transgenic Res. 2025 Jun 16;34(1):30. doi: 10.1007/s11248-025-00449-6 (PMC12170776; doi:10.1007/s11248-025-00449-6)
Supplement: Supplementary file 3 — Supplementary file3 (PPTX 143 kb) [file 11248_2025_449_MOESM3_ESM.pptx]

## Slide 1
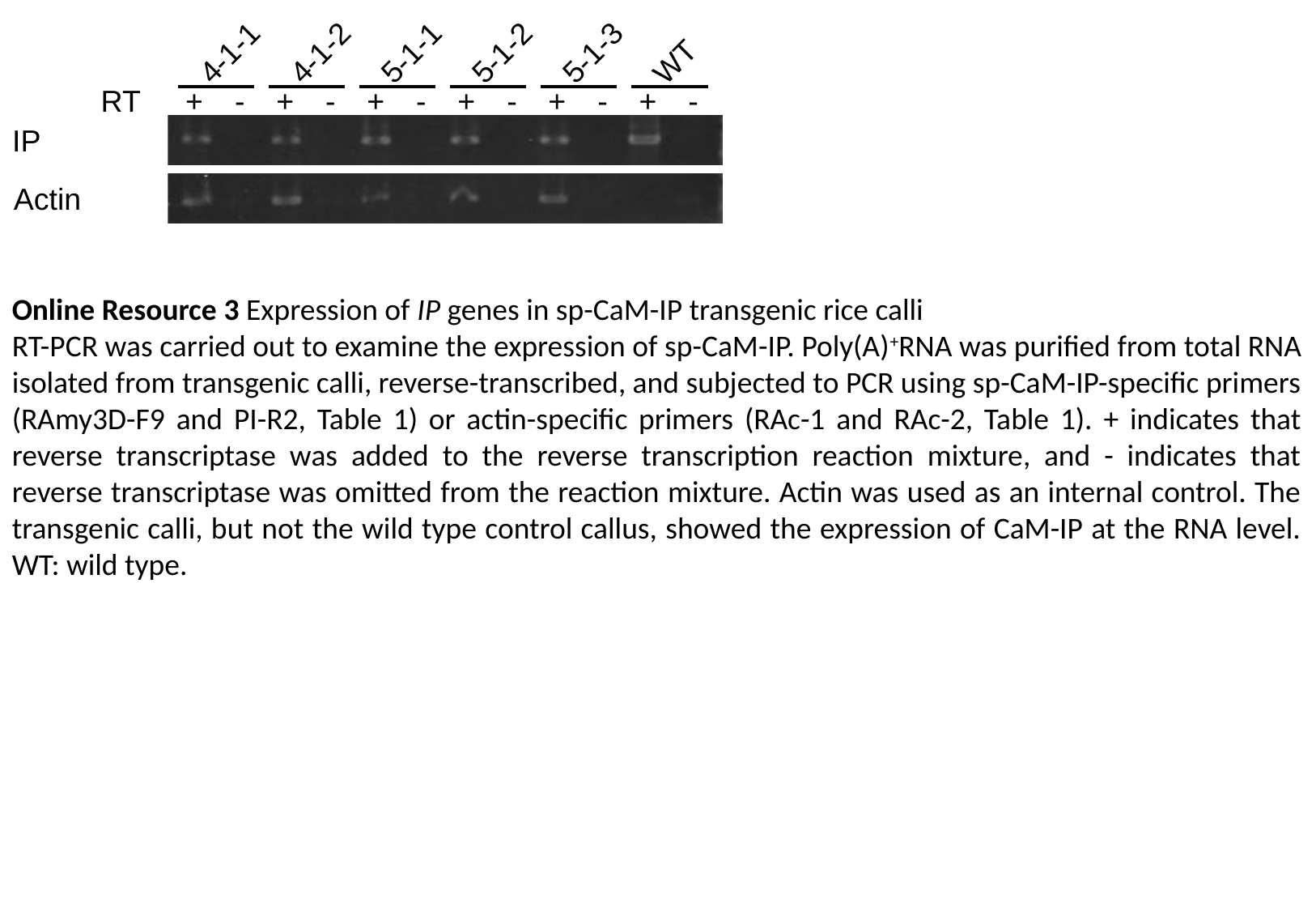

4-1-1
4-1-2
5-1-1
5-1-2
5-1-3
WT
+
-
+
-
+
-
+
-
+
-
+
-
RT
IP
Actin
Online Resource 3 Expression of IP genes in sp-CaM-IP transgenic rice calli
RT-PCR was carried out to examine the expression of sp-CaM-IP. Poly(A)+RNA was purified from total RNA isolated from transgenic calli, reverse-transcribed, and subjected to PCR using sp-CaM-IP-specific primers (RAmy3D-F9 and PI-R2, Table 1) or actin-specific primers (RAc-1 and RAc-2, Table 1). + indicates that reverse transcriptase was added to the reverse transcription reaction mixture, and - indicates that reverse transcriptase was omitted from the reaction mixture. Actin was used as an internal control. The transgenic calli, but not the wild type control callus, showed the expression of CaM-IP at the RNA level. WT: wild type.
