# Supplementary material for: Expression of an antimicrobial peptide persulcatusin fused with calmodulin in rice cultured cells
Source: Transgenic Res. 2025 Jun 16;34(1):30. doi: 10.1007/s11248-025-00449-6 (PMC12170776; doi:10.1007/s11248-025-00449-6)
Supplement: Supplementary file 4 — Supplementary file4 (PPTX 1109 kb) [file 11248_2025_449_MOESM4_ESM.pptx]

## Slide 1
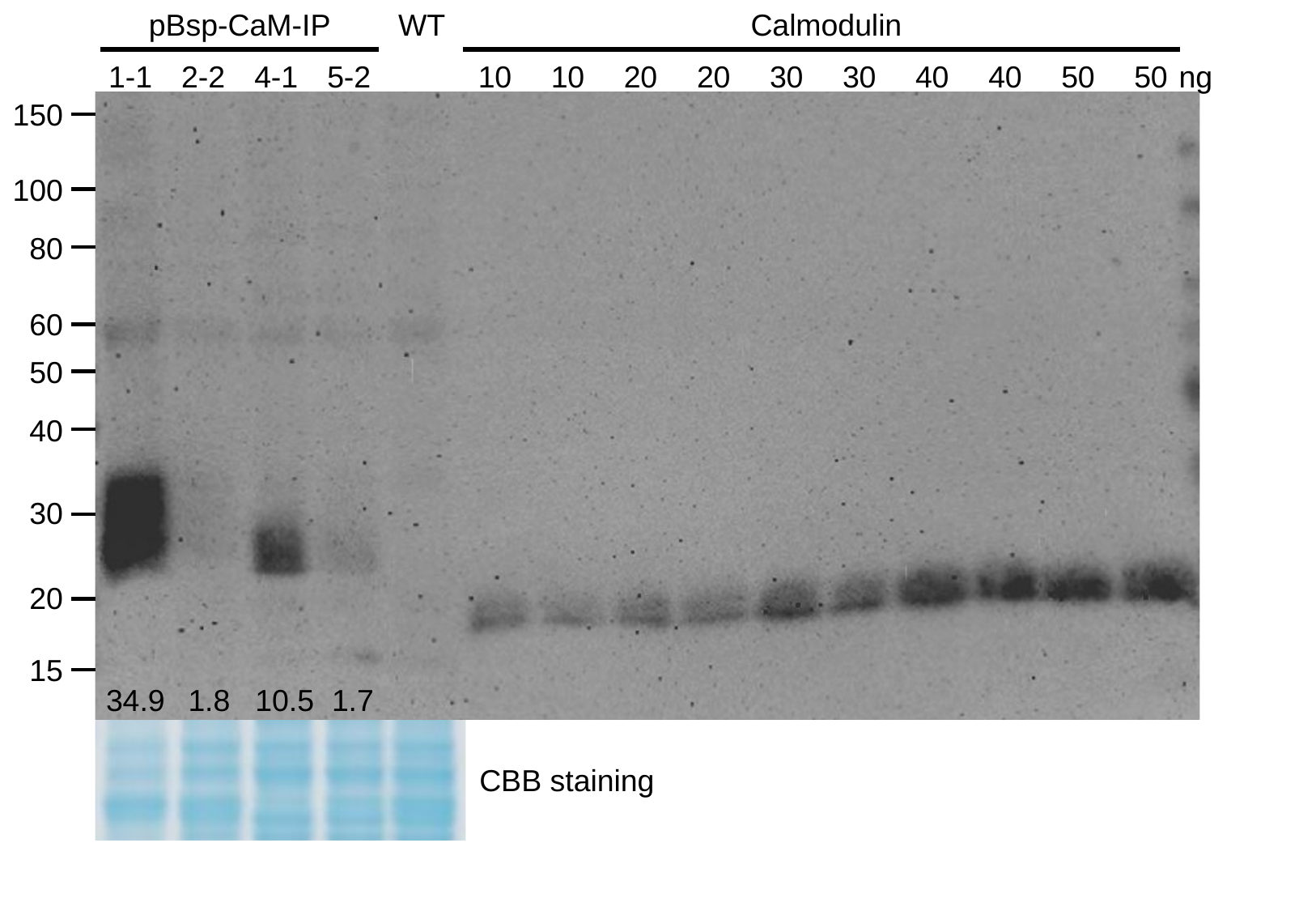

pBsp-CaM-IP
WT
Calmodulin
1-1
2-2
4-1
5-2
10
10
20
20
30
30
40
40
50
50
ng
150
100
80
60
50
40
30
20
15
34.9
1.8
10.5
1.7
CBB staining

## Slide 2
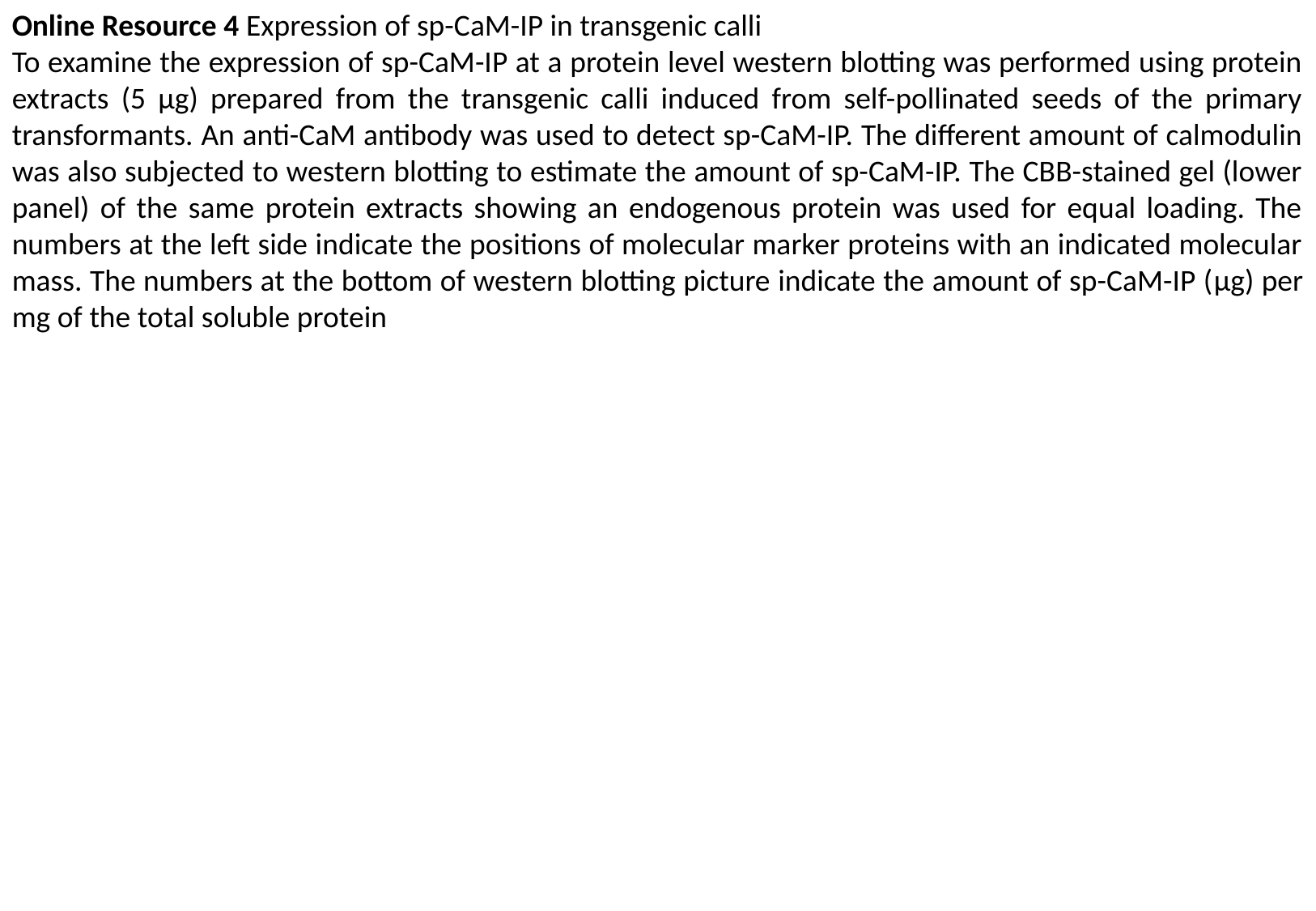

Online Resource 4 Expression of sp-CaM-IP in transgenic calli
To examine the expression of sp-CaM-IP at a protein level western blotting was performed using protein extracts (5 μg) prepared from the transgenic calli induced from self-pollinated seeds of the primary transformants. An anti-CaM antibody was used to detect sp-CaM-IP. The different amount of calmodulin was also subjected to western blotting to estimate the amount of sp-CaM-IP. The CBB-stained gel (lower panel) of the same protein extracts showing an endogenous protein was used for equal loading. The numbers at the left side indicate the positions of molecular marker proteins with an indicated molecular mass. The numbers at the bottom of western blotting picture indicate the amount of sp-CaM-IP (μg) per mg of the total soluble protein
