# Supplementary material for: Expression of an antimicrobial peptide persulcatusin fused with calmodulin in rice cultured cells
Source: Transgenic Res. 2025 Jun 16;34(1):30. doi: 10.1007/s11248-025-00449-6 (PMC12170776; doi:10.1007/s11248-025-00449-6)
Supplement: Supplementary file 5 — Supplementary file5 (PPTX 226 kb) [file 11248_2025_449_MOESM5_ESM.pptx]

## Slide 1
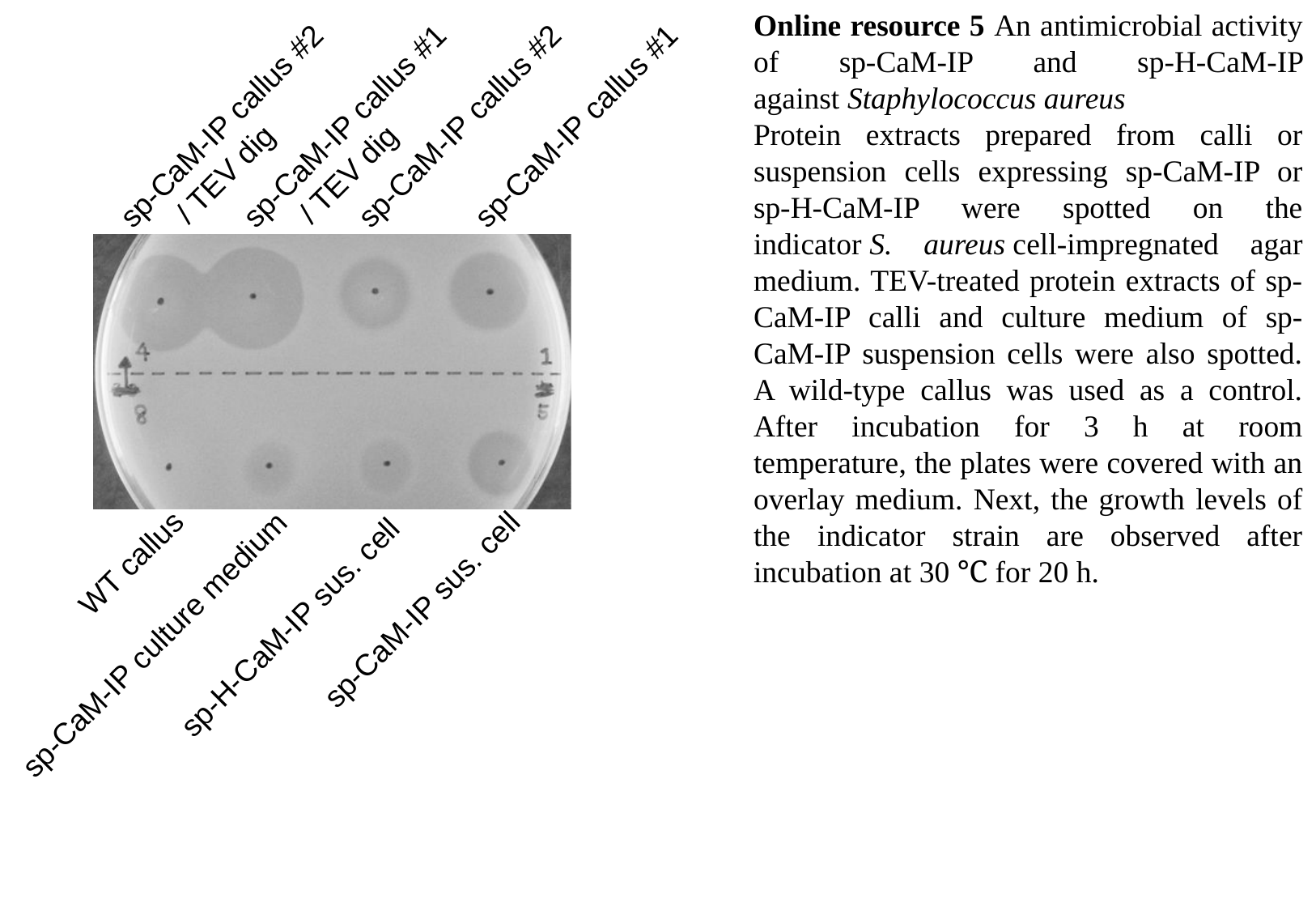

Online resource 5 An antimicrobial activity of sp-CaM-IP and sp-H-CaM-IP against Staphylococcus aureus
Protein extracts prepared from calli or suspension cells expressing sp-CaM-IP or sp-H-CaM-IP were spotted on the indicator S. aureus cell-impregnated agar medium. TEV-treated protein extracts of sp-CaM-IP calli and culture medium of sp-CaM-IP suspension cells were also spotted. A wild-type callus was used as a control. After incubation for 3 h at room temperature, the plates were covered with an overlay medium. Next, the growth levels of the indicator strain are observed after incubation at 30 ℃ for 20 h.
sp-CaM-IP callus #2
 / TEV dig
sp-CaM-IP callus #1
 / TEV dig
sp-CaM-IP callus #2
sp-CaM-IP callus #1
WT callus
sp-CaM-IP sus. cell
sp-H-CaM-IP sus. cell
sp-CaM-IP culture medium
