# Supplementary material for: Expression of an antimicrobial peptide persulcatusin fused with calmodulin in rice cultured cells
Source: Transgenic Res. 2025 Jun 16;34(1):30. doi: 10.1007/s11248-025-00449-6 (PMC12170776; doi:10.1007/s11248-025-00449-6)
Supplement: Supplementary file 6 — Supplementary file6 (PPTX 1247 kb) [file 11248_2025_449_MOESM6_ESM.pptx]

## Slide 1
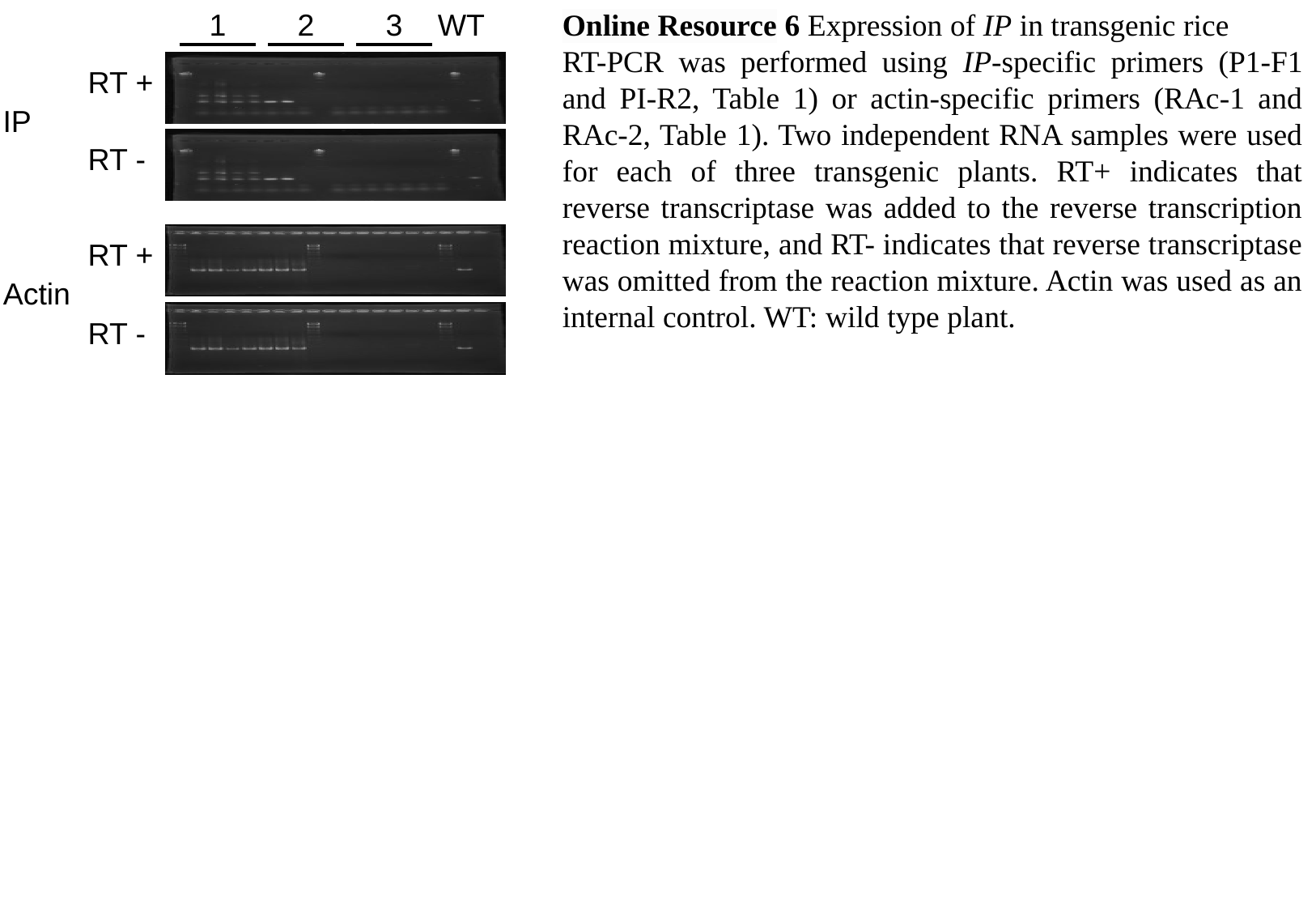

1
2
3
WT
Online Resource 6 Expression of IP in transgenic rice
RT-PCR was performed using IP-specific primers (P1-F1 and PI-R2, Table 1) or actin-specific primers (RAc-1 and RAc-2, Table 1). Two independent RNA samples were used for each of three transgenic plants. RT+ indicates that reverse transcriptase was added to the reverse transcription reaction mixture, and RT- indicates that reverse transcriptase was omitted from the reaction mixture. Actin was used as an internal control. WT: wild type plant.
RT +
IP
RT -
RT +
Actin
RT -
