# Supplementary material for: Expression of an antimicrobial peptide persulcatusin fused with calmodulin in rice cultured cells
Source: Transgenic Res. 2025 Jun 16;34(1):30. doi: 10.1007/s11248-025-00449-6 (PMC12170776; doi:10.1007/s11248-025-00449-6)
Supplement: Supplementary file 7 — Supplementary file7 (PPTX 9550 kb) [file 11248_2025_449_MOESM7_ESM.pptx]

## Slide 1
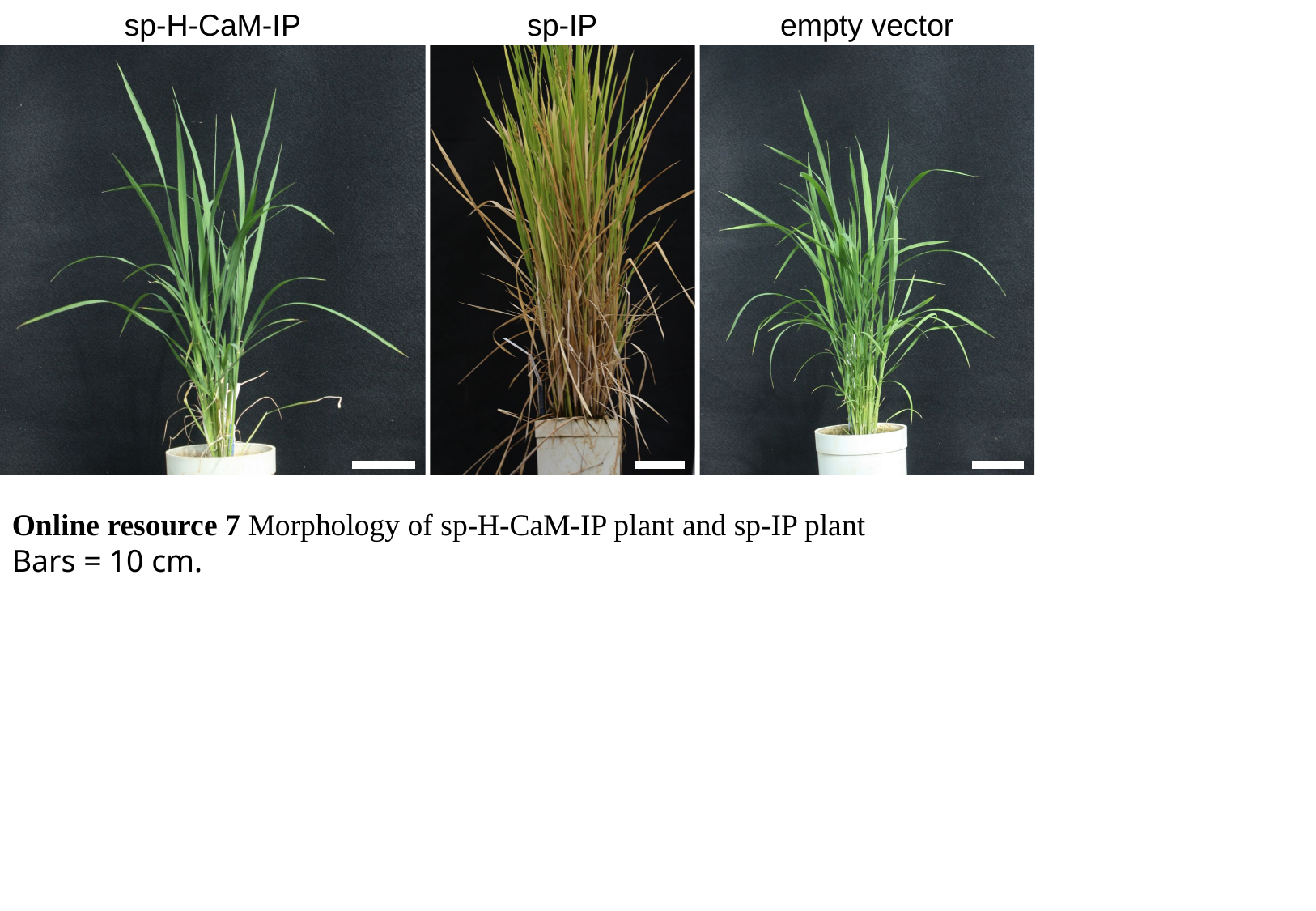

sp-H-CaM-IP
sp-IP
empty vector
Online resource 7 Morphology of sp-H-CaM-IP plant and sp-IP plant
Bars = 10 cm.
